# Nodal promotes colorectal cancer survival and metastasis through regulating SCD1-mediated ferroptosis resistance

**DOI:** 10.1038/s41419-023-05756-6

**Published:** 2023-03-31

**Authors:** Tianqi Wu, Jian Wan, Xiao Qu, Kai Xia, Fangtao Wang, Zichao Zhang, Muqing Yang, Xiaocai Wu, Renyuan Gao, Xiaoqi Yuan, Lin Fang, Chunqiu Chen, Lu Yin

**Affiliations:** 1 Center for Difficult and Complicated Abdominal Surgery, Shanghai Tenth People’s Hospital, Tongji University School of Medicine, Shanghai, 200072 China; 2 Department of Hepatobiliary and Pancreatic Surgery, Affiliated Hospital of Nantong University, Nantong, 226000 China; 3 Department of Breast and Thyroid Surgery, Shanghai Tenth People’s Hospital, Tongji University School of Medicine, Shanghai, 200072 China

**Keywords:** Colorectal cancer

## Abstract

Re-expression of an embryonic morphogen, Nodal, has been seen in several types of malignant tumours. By far, studies about Nodal’s role in colorectal cancer (CRC) remain limited. Ferroptosis is essential for CRC progression, which is caused by cellular redox imbalance and characterized by lipid peroxidation. Herein, we observed that Nodal enhanced CRC cell’s proliferative rate, motility, invasiveness, and epithelial–mesenchymal transition (EMT) in vivo and in vitro. Notably, Nodal overexpression induced monounsaturated fatty acids synthesis and increased the lipid unsaturation level. Nodal knockdown resulted in increased CRC cell lipid peroxidation. Stearoyl-coenzyme A desaturase 1 (SCD1) inhibition at least partially abolished the resistance of Nodal-overexpressing cells to RSL3-induced ferroptosis. Mechanistically, SCD1 was transcriptionally up-regulated by Smad2/3 pathway activation in response to Nodal overexpression. Significant Nodal and SCD1 up-regulation were observed in CRC tissues and were associated with CRC metastasis and poor clinical outcomes. Furthermore, bovine serum albumin nanoparticles/si-Nodal nanocomplexes targeting Nodal had anti-tumour effects on CRC progression and metastasis. This research elucidated the role of Nodal in CRC development and revealed a potential gene-based therapeutic strategy targeting Nodal for improving CRC treatment.

## Introduction

Colorectal cancer (CRC) is a primary contributor to cancer-related mortality globally [1]. Patients diagnosed with advanced-stage metastatic CRC have a dismal 5-year survival rate [2]. Although novel therapeutic targets for CRC have been identified, the mechanisms underlying CRC metastasis remain unclear owing to biological heterogeneity.

Similar cancer hallmarks are shared between cancer metastasis and embryonic stem cell development. The embryonic stem cell-associated genes are more frequently overexpressed in aggressive cancers [3], which is associated with cancer metastasis and poor prognosis [4]. One of the significant embryonic stem cell-associated genes is Nodal. Nodal belongs to the transforming growth factor (TGF) superfamily and is associated with embryonic stem cell differentiation and development [5]. Nodal binds to its cell-surface receptors and exerts its biological effects by activating the intracellular Smad2/3 signalling pathway [6].

Nodal is not expressed in normal tissues; however, it is re-expressed in invasive epithelial-derived malignancies [7]. It is speculated that re-emerging Nodal promotes cancer development and invasion, as well as mediates normal invasive events in embryonic development. Reportedly, Nodal facilitates cell invasion and angiogenesis in melanoma, prostate cancer, and breast cancer [8, 9]. Nodal signalling inhibition improves the efficacy of drug therapy in pancreatic cancer [10]. Major histocompatibility complex class I chain-related protein A expression might be impacted by Nodal in the tumour microenvironment, thereby allowing γδT cells to mediate immune escape [11]. Nodal performs an instrumental function in modulating cancer cell stemness in CRC [12, 13]. Recent studies have demonstrated more varied functions of Nodal. One study reported that Nodal inhibits oxidative stress during cerebral ischaemia-reperfusion injury [14]. However, whether Nodal regulates oxidative stress in malignancy development has not been demonstrated.

Carcinogenesis and metastasis depend on redox homeostasis. Few cancer cells survive or even proliferate in the circulation during metastasis, making it an ineffective process. Reportedly, ferroptosis might be responsible for the limited survival of cancer cells in the circulation [15]. Ferroptosis, a type of iron-dependent cell death, is correlated with elevated levels of lipid peroxidation that were triggered by reactive oxygen species (ROS). Targeting ferroptosis-related molecules might offer tremendous potential for treating CRC [16]. RSL3 induces ferroptosis in CRC, while glutathione peroxidase 4 effectively reverses the effect [17]. Cetuximab promotes ferroptosis by suppressing the nuclear factor erythroid 2–related factor 2/heme oxygenase 1 signalling pathway in KRAS mutant CRC cells, which has been recently demonstrated in nude mouse models [18]. Ferroptosis sensitivity depends on the fatty acid (FA) balance. Polyunsaturated FAs (PUFAs) promote free radical production and lipid peroxide accumulation, thereby triggering ferroptosis [19]. PUFA biosynthesis enzyme inhibition increases cell resistance to ferroptosis, which can be reversed by the addition of exogenous PUFAs [20]. Stearoyl-coenzyme A desaturase 1 (SCD1), a lipid-modifying enzyme, catalyses saturated FA desaturation to monounsaturated FAs (MUFAs), which could prevent lipid peroxide accumulation by replacing PUFAs in the plasma membrane and further inducing ferroptosis resistance [21]. It is up-regulated in several malignancies [22] and has been demonstrated as a key target of several pathways, including the 5’ adenosine monophosphate-activated protein kinase pathway and phosphoinositide 3-kinase-protein kinase B-mammalian target of rapamycin pathway, by which ferroptosis resistance is triggered in cancer cells [23, 24]. However, the potential significance and regulatory mechanism of SCD1 and ferroptosis in CRC progression and metastasis are not completely elucidated.

RNA interference (RNAi) refers to inhibited target gene expression by specific small interfering RNAs (siRNAs). However, siRNA’s high degradability and immunogenicity limit its clinical applicability. According to recent studies, the high biocompatibility and efficiency of bovine serum albumin nanoparticles (BSA-NPs) render them to be well-established siRNA carriers, indicating a promising potential for using RNAi in cancer treatment [25].

Herein, we demonstrated Nodal overexpression in CRC, which was associated with CRC progression and metastasis. More importantly, Nodal induced ferroptosis resistance and promoted cancer cell survival and metastasis by up-regulating downstream SCD1. Gene therapy targeting Nodal was found to be highly effective in vivo and in vitro for treating CRC, thereby highlighting the translational potential of Nodal as a new strategy for CRC therapy.

## Methods and Materials

### Reagents and antibodies

RSL3 (HY-100218, MedChemExpress), chloroquine (CQ) (HY-17589A, MedChemExpress), necrostatin-1 (Nec-1) (HY-15760, MedChemExpress), ferrostatin-1 (Fer-1) (HY-100579, MedChemExpress), SB431542 (HY-10431, MedChemExpress), and carbon 11 (C11)-BODIPY^581/591^ (RM02821, Abclonal, Wuhan, China) were the reagents used.

Nodal (A9902, Abclonal; sc-373910, Santa Cruz Biotechnology), SCD1 (ab236868, Abcam), Phospho-Smad2 (Ser465/467)/Smad3 (Ser423/425) (8828 S, Cell Signalling Technology), Smad2/3 (ab202445, Abcam; 5678, Cell Signalling Technology), E-cadherin (A20798, Abclonal), N-cadherin (A0432, Abclonal), vimentin (ab8978, Abcam), snail (A5243, Abclonal), GAPDH (A19056, Abclonal), β-tubulin (AC008, Abclonal), goat anti-rabbit immunoglobulin (Ig) G heavy and light chain (H&L) (horse radish peroxidase [HRP]) (ab6721, Abcam), goat anti-mouse IgG (H&L) (HRP) (ab6789, Abcam), IRDye 680 donkey anti-mouse IgG-(H + L)/goat anti-rabbit IRDye 800CW secondary antibody (926-68072/926-32211, LI-COR Biosciences, Lincoln, NE, USA) were the antibodies used in this study.

### Patients

We obtained 75 CRC tissues and adjacent non-tumour tissues with the corresponding clinical information of patients from the Centre for Difficult and Complicated Abdominal Surgery, Shanghai Tenth People’s Hospital of Tongji University (Shanghai, China). The sample sizes were large enough to measure the effect size. Histopathological analysis was performed to verify all samples, and preoperative chemotherapy was not administered to any of the participants. The Institutional Ethics Committees of Shanghai Tenth People’s Hospital (SHSY-IEC-4.1/21-228/01) approved this study, and patients or their relatives granted their written consent after being fully informed about the study. The principles outlined in the Declaration of Helsinki were adhered to during this research. The data linked to patients’ clinicopathological conditions are shown in Table 1.

**Table 1:** Association of Nodal expression with clinicopathological data from patients.

Characteristics	Patient		Nodal expression	
	Total	Low	High	*p*-value
**Gender**				0.12
Male	48	11	37	
Female	27	11	16	
**Age (y)**				0.794
≤ 60	28	9	19	
> 60	47	13	34	
**Tumour location**				0.309
Colon	43	10	32	
Rectum	32	12	21	
**Tumour size (cm)**				**0.001**
≤ 5	51	21	30	
> 5	24	1	23	
**Tumour differentiation**				0.264
Well / moderate	65	21	44	
Poor	10	1	9	
**Tumour invasion**				**<** **0.0001**
T1 + T2	15	12	3	
T3 + T4	60	10	50	
**Lymph node metastasis**				**<** **0.0001**
Negative	34	22	12	
Positive	41	0	41	
**Distant metastasis**				**0.0017**
Negative	58	22	36	
Positive	17	0	17	

### Cell lines

The SW480, SW620, HCT116, Caco2, and Lovo cell lines were supplied by the Cell Bank of the Chinese Academy of Sciences (Shanghai, China), where short tandem repeat analysis was conducted for authentication. All cells were grown in a humid environment of 5% CO_2_ at 37 °C in Dulbecco’s Modified Eagle’s Medium (DMEM) (Gibco, USA) supplemented with 10% foetal bovine serum (FBS) (Gibco, USA), streptomycin (100 μg/mL) (Enpromise, China), and penicillin (100 units/mL) (Enpromise, China).

### Plasmid construction and transfection

GENERAY Biotechnology (Shanghai, China) synthesised the siRNA targeting Nodal (si-Nodal), si-SCD1, the negative control, and SCD1 overexpression plasmid. Table S1 contains a list of all of the siRNA sequences. Lipofectamine® 3000 (Invitrogen; Thermo Fisher Scientific, USA) was employed for the transfection of siRNA and plasmid as directed by the manufacturer. The Nodal lentiviral overexpression construct and control vector were supplied by ZORIN (Shanghai, China), and transfection was done as mentioned above. Subsequently, selective puromycin resistance served as the basis for isolating stably overexpressed cells and control cells.

### RNA isolation, quantitative polymerase chain reaction (qPCR), and reverse transcription (RT)-PCR

Following the protocol provided by the manufacturer, total RNA was isolated from the frozen tissues or treated cells utilising the Trizol reagent (manufactured by Invitrogen and located in Carlsbad, California, United States). Purified RNA samples were analysed with a Nanodrop 2000 spectrophotometer (Thermo Fisher Scientific, USA). Synthesis of cDNA was accomplished with the use of a commercially available cDNA synthesis kit (Takara Biotechnology, Dalian, China), and the SYBR Green PCR kit (Takara Biotechnology, Dalian, China) was applied to evaluate the target gene expression. Subsequently, the relative expression levels were derived utilising the 2^−△△CT^ method. The primers synthesised and desalted by GENERAY Biotechnology (Shanghai, China) are displayed in Table S1.

### Cell viability assay

To assess the viability of the cells, a Cell Counting Kit-8 (CCK-8) (Yeasen, Shanghai, China) was employed. Then, 96-well plates were seeded with (1 × 10^3^) CRC cells. Each well was injected with 10 μL of the CCK-8 reagent. After incubating the cells for 1, 2, 3, 4, and 5 days, they were kept in a 37 °C incubator for 2 h. Subsequently, the absorbance was assessed by utilising a microplate spectrophotometer at 450 nm (BioTek, Instruments, Inc., Winooski, VT, USA). Furthermore, 96-well plates were employed to seed the cells before treating them with relevant reagents after 2 days of culture to analyse the cytotoxicity and detect the ferroptosis resistance. The absorbance was measured after 48 h of treatment according to the above-mentioned procedure. Untreated cells with a survival rate of 100% served as the controls and the survival rate of the other cell groups was calculated based on that of the control group.

### Colony formation assay

Six-well plates were seeded with CRC cells (density: 500 per well), and the cells were cultured for 2 weeks. These samples were rinsed using phosphate-buffered saline (PBS), following which they were stained with 0.1% crystal violet for half an hour after fixing them in 75% ethanol. Thereafter, tumour cell colonies were imaged and counted manually.

### Wound healing assay

Six-well plates were used to seed the CRC cells. When the treated cells reached approximately 80% confluence, 200 μL pipette tips were used to generate the “wounds”, and 2% FBS was added to DMEM used for cell culture. A light microscope was used to assess wound healing, and images were captured at 0, 24, and 48 h at the same position. The migratory rate was determined by using the following formula: (1 − distance at other time points/distance at 0 h) × 100%.

### Transwell assay

CRC cells (8 × 10^4^) were added to a Transwell chamber (pores measuring 8 μm in size, Corning Inc., Lowell, MA, USA) coated with matrigel (to analyse invasion rather than a migration; BD Biosciences, Franklin Lakes, NJ, USA). After that, 200 μL of a serum-free medium (SFM) with cells was introduced to the upper chamber, and the lower chamber was introduced with 500 μL of a medium comprising 10% FBS. Following 20–48 h of incubation, the cells in the upper chamber were removed, whereas those in the lower chamber were fixed in 70% ethanol, followed by staining with 0.1% crystal violet. Subsequently, representative images were taken at × 200 magnification with a light microscope (Olympus Corporation, Tokyo, Japan), and cells in six fields per filter were randomly counted. Data are expressed as migrated/invaded cells per field.

### Western blotting

Extraction of total protein from frozen tissues or treated CRC cells was done utilising RIPA lysis buffer (Beyotime, Jiangsu, China). Thereafter, the protein concentration was assessed utilising a BCA protein assay kit (Beyotime, Jiangsu, China). For separation, an equivalent amount of protein was loaded onto 8–10% sodium dodecyl sulphate-polyacrylamide gel before transferring the isolated proteins onto 0.45 μm nitrocellulose membranes (Beyotime). Five percent non-fat milk in PBS was employed to block the membranes for 1 h at room temperature (RT), followed by incubation at 4 °C throughout the night with primary antibodies diluted in a blocking buffer. Following a three-time washing procedure, corresponding secondary antibodies were introduced into the membranes and subjected to incubation for 1 h at RT. An Odyssey Scanning system (LI-COR Biosciences, Lincoln, NE, USA) or a Tanon (Shanghai, China) chemiluminescence image analysis system was used to analyse the protein bands.

### Immunohistochemical (IHC) analysis

IHC analysis was performed on 5 μm sections of samples fixed in formalin and embedded in paraffin. The sections were dewaxed using xylene and rehydrated using graded concentrations of ethanol. We utilised a citrate antigen repair solution of 10 nM (Sangon Biotech; Shanghai, China) and a microwave oven set to 95 °C for 10 min to retrieve antigens, and hydrogen peroxide at a concentration of 3% was employed to block the activity of endogenous peroxidase. Later, primary antibodies were used to wash and incubate the sections overnight at 4 °C.

### Immunofluorescence (IF) analysis

Paraffin sections were deparaffinised, dehydrated with graded concentrations of alcohol, and subjected to antigen retrieval for IF analysis. Five percent BSA was applied to block the sections at RT for half an hour before incubating them at 4 °C throughout the night with a primary antibody. Afterward, the corresponding fluorescent-dye-labelled secondary antibodies were used to incubate the slides at RT for 1 h. An Olympus fluorescence microscope was used to observe the slides, following which they were photographed.

### C11-BODIPY

Flow cytometry was performed by seeding the cells in a 6-well plate (Corning), treating them with 5 μM DMSO or RSL3 for 48 h, and subsequently incubating them with 5 μM C11-BODIPY for 1 h. Next, the cells were dissociated, rinsed, and resuspended, following which a flow cytometer was used to evaluate the fluorescence intensity (FACSCanto™ II; BD Biosciences). Fluorescence channel (FL) 1 (excitation, 488 nm) was used to measure the oxidised lipid, whereas FL2 (excitation, 561 nm) was used to measure the unoxidised oxidised lipid. The fluorescence intensity ratio of FL1 to FL2 was used to quantify the proportion of peroxidised lipid cells.

Confocal imaging was performed by seeding the cells in confocal dishes with or without RSL3 for 48 h and subsequently incubating them for one hour with 5 μM C11-BODIPY. Thereafter, 4% paraformaldehyde was employed to wash and fix the cells, and an inverted microscope (Carl Zeiss, LSM 900) was used to capture images.

### Malondialdehyde (MDA) assay

The cell preparation for the MDA assay was similar to that for C11-BODIPY staining. After cell homogenisation, the MDA concentration was detected using a lipid peroxidation (MDA) assay kit (KTB1050, Abbkine, Wuhan, China) per the guidelines stipulated by the manufacturer. In brief, the MDA in the sample underwent a reaction with thiobarbituric acid (TBA), resulting in the formation of MDA-TBA adducts which were subsequently quantified utilising a colourimetry at an absorbance of 532 nm. Additionally, the absorbance was detected at 600 nm to eliminate sucrose interference. Furthermore, the MDA levels in the reaction system were evaluated by measuring the difference in absorbance between 532 and 600 nm based on the standard MDA curve, and the MDA content in cells was calculated based on the protein concentration of the samples.

### Transmission electron microscopy (TEM)

HCT116 cells were seeded in a 10 cm^2^ cell dish and pre-treated with relevant reagents. Subsequently, the cells were collected, and 2.5% glutaraldehyde was used to fix the cells. Transmission electron microscopy (TEM) was conducted by Servicebio (Wuhan, China).

### Dual-luciferase reporter assay

A wild-type pGL3-basic-SCD1 promoter plasmid was transfected into HCT116 cells. Then, to investigate the impact of the Nodal-activated Smad2/3 pathway on the transcriptional activity of SCD1, the cells were exposed to varying concentrations of SB431542 before being analysed. A Dual-Luciferase Reporter Assay kit (MA0518, Meilun Biotechnology, China) was employed in evaluating the transcriptional activity of SCD1, and calculations were made to determine the ratio of firefly to Renilla luciferase activity. In addition, several pGL3-basic-SCD1 promoter plasmids (wild-type, mutant-type, and truncated-type) were co-transfected with pcDNA3.1-NC or pcDNA3.1-Nodal plasmids into 293T and HCT116 cells, and the luciferase activity was detected after 24 h. IBSBio (Shanghai, China) designed and synthesised all the pGL3-basic-SCD1 plasmids. The pcDNA3.1-NC or pcDNA3.1-Nodal plasmids were procured from ZORIN (Shanghai, China).

### Chromatin immunoprecipitation (ChIP)

CRC cells (1 × 10^7^) were harvested for ChIP assay. After cross-linking the cells for 10 min in 1% formaldehyde at 37 °C, they were rinsed in PBS before resuspension in 300 μL of lysis buffer. Sonication was used to break down the DNA into smaller fragments, and ChIP-grade Smad2/3 or IgG (NC) antibodies were used to immunoprecipitate the fragmented DNA. Subsequently, SYBR green-based qPCR was used to amplify the immunoprecipitated DNA using primers encompassing the corresponding binding sites in the promoter region of SCD1.

### Preparation of BSA-NPs

As previously described, the desolvation-crosslinking method was used to synthesise BSA-NPs and BSA-NPs/siRNA [25]. Briefly, BSA (50 mg) was dissolved in 2 mL of sodium chloride solution (10 mM). This BSA solution was then vigorously stirred for 15 min using magnetic stirring (500 rpm) at RT. After the BSA solution’s pH was adjusted to 9.0, ethanol was gradually introduced to the BSA solution dropwise until the solution became turbid with constant stirring. Thereafter, to produce BSA-NPs, the NP solution was stirred for three hours at RT, followed by the addition of 14 μL of 2.5% glutaraldehyde aqueous solution. After stirring the mixture for a total of twenty-four hours at RT, the BSA-NP suspension was centrifuged three times (12,000 × g, 30 min) to achieve purity, and the pellets obtained were resuspended in deionised water.

### Preparation of BSA-NP/siRNA and agarose gel retardation assay

BSA (20 mg) was dissolved in sodium chloride solution (1 mL, 10 mM) and stirred for 15 min to synthesise BSA-NP/siRNA complexes. Subsequently, the siRNA for Nodal was added to BSA, and the mixture was stirred for 1 h at RT. The solution’s pH was adjusted to 9.0, and BSA-NP/siRNA complexes were obtained by following the protocol for BSA-NP preparation.

The loading of siRNAs onto BSA-NPs was quantified using the agarose gel retardation assay. Briefly, complexes of BSA-NP/siRNA were produced using a variety of various weight-to-weight (w/w) ratios (1:5, 1:2, 1:1, 1:0.9, 1:0.8, and 1:0.5). A total of 0.375 g of agarose was added to 25 mL of 1 × Tris base, acetic acid and ethylenediaminetetraacetic acid buffer, following which 2.5 μL of the GoldView staining solution was added to the mixture. After the agarose solidified, BSA-NP/siRNA supernatants of different w/w ratios were collected, mixed with the DNA loading buffer, and subjected to electrophoresis at a constant voltage of 130 V for 20 min. The free siRNA served as the control. A Tanon gel imaging system (Shanghai, China) was used to capture images.

### Characterisation of BSA-NP/siRNA

Deionised water (BSA, 1 mg/mL) was employed to dilute the BSA-NP/siRNA complex. To determine the mean hydrodynamic droplet size of the BSA-NP/siRNA complex, dynamic light scattering (Zetasizer 3000 HS, Malvern Instruments Ltd., UK) with a 633 nm helium-neon laser at a scattering angle of 90° was used. At 25 °C, the electrophoretic mobility was measured with the same equipment, and this allowed for the determination of the complex’s zeta potential, and the surface morphology was observed via TEM (Hitachi HT7700, Japan).

### RNA-sequencing (RNA-Seq)

Trizol (Invitrogen, Carlsbad, CA, USA) was utilised for total RNA isolation from empty vector-transfected HCT116 cells (vector-HCT116 cells) and Nodal-overexpressing HCT116 cells (oe-Nodal-HCT116 cells). The BGIseq500 platform (Beijing Genomics Institute (BGI)-Shenzhen, China) was then used for further RNA processing, development of the library, and sequence analysis. After acquiring sequencing data, standard bioinformatic analyses were performed by the BGI. Gene expression was evaluated using RNA-Seq by Expectation-Maximisation (v1.2.12), and Pheatmap (v1.0.8) was used to plot the heatmap. Based on a Q-value of ≤ 0.05, the groups were screened for differentially expressed genes (DEGs) using DESeq2 (v1.4.5). These DEGs were further annotated via the Kyoto Encyclopaedia of Genes and Genomes (KEGG) enrichment analysis (https://www.kegg.jp/). The experiment was performed in triplicate. The data were deposited in a public database (PRJNA944601).

### Ultra-performance liquid chromatography-tandem mass spectrometry (UPLC-MS/MS)

Shanghai Lu-Ming Biotech Co., Ltd. (Shanghai, China) analysed the targeted cell metabolites. First, after preparing a standard stock solution (MSS) and diluting it, calibration curves were drawn. Samples comprising the medium (PBS) were volatilised and treated for subsequent UPLC-MS/MS analysis. Liquid chromatography was conducted on a Nexera ultra-high-performance liquid chromatography LC-30A system (SHIMADZU) using a Waters Acquity UPLC bridged ethyl hybrid C18 column (2.1 × 100 mm, 1.7 μm) at 50 °C. Mobile phase A was water, which comprised 0.05% formic acid, whereas mobile phase B was acetonitirle:2-propanol (9:1, volume/volume). Next, mass spectrometry was carried out using an AB SCIEX Selex ION Triple Quad^TM^ 5500 system in a negative ion mode using an electrospray ionization source. Targeted metabolites were analysed in the multiple-reaction monitoring (MRM) mode. The Analyst was used for data acquisition and further analysis, and SCIEX OS-MQ was used to quantify the identified metabolites.

### Xenograft tumour models

The animal experiments conducted in this study complied with the applicable ethical regulations approved by the Committee on the Ethics of Animal Experiments of Shanghai Tenth People’s Hospital Affiliated with Tongji University. In particular, female BALB/c nude mice (age, 4 weeks) were procured from Shanghai Slack Laboratory Animals Co., Ltd. (Shanghai, China). The mice were kept in a specific pathogen-free room and provided with an ad libitum supply of sterilised water and food. Subsequently, the mice were classified at random into two groups as per their body weight. The two groups were as follows: the control group (mice injected with vector-HCT116 cells); the overexpression group (mice injected with oe-Nodal-HCT116 cells). All animal experiments were conducted in a double-blinded manner.

The CRC xenograft model was developed by subcutaneously injecting 5 × 10^6^ HCT116 cells in 0.1 mL of PBS, into the right axilla of each mouse. A vernier calliper was used to measure the tumour volume every 3 days, and the volume of the tumour was determined based on the below equation: 0.5 × length × width^2^. After 30 days, they were euthanised, and the tumour weight and volume were measured. The harvested tumours were fixed with formalin and prepared for detection.

A metastatic model was developed by injecting the female BALB/c nude mice with 1 × 10^6^ luciferase-labelled cells into their tail vein. The mice were anaesthetised and intraperitoneally injected with potassium fluorescein salt (150 mg/kg) after 4 weeks. An IVIS imaging system (Calipers, Hopkinton, USA) was used to identify and photograph tumours, and the luminescence intensity of metastatic nodules with lung metastases was calculated.

The above-mentioned tumour models were established by inoculating nude mice with oe-Nodal-HCT116 cells to evaluate the therapeutic effects of BSA-NP/si-Nodal in vivo. After the volume of subcutaneous tumours reached approximately 100 mm^3^ and lung metastasis was observed via bioluminescence imaging, the mice were classified at random into three groups as follows: mice treated with intratumoural or intravenous PBS, those treated with free si-Nodal (5 nmol), and those treated with BSA-NP/si-Nodal (5 nmol) once every 3 days for 15 days. Subsequently, we evaluated the tumour weight and volume, and lung metastasis was examined as described above. After sacrificing the mice, the vital organs (the kidneys, spleen, lungs, liver, and heart) were collected for biosafety evaluation.

### Statistical analysis

The analyses of statistical data were conducted with GraphPad Prism (version 9.2.0). All data are presented as the mean ± standard deviation. Differences (variations) between groups were examined utilising the two-tailed Student’s t-test or one-way analysis of variance. Survival curves were generated utilising the Kaplan-Meier approach, and a comparative assessment of survival between groups was accomplished with the log-rank test. The correlation between two variables was estimated through Pearson’s correlation analysis. There were three separate runs of each experiment. The criterion for significance was determined to be *P* ≤ 0.05 (* denotes *P*-values of <0.05, ** denotes *P*-values of <0.01, and *** denotes *P*-values of < 0.001).

### Illustration tool

BioRender (https://biorender.com/) was used to create the graphical abstract images.

## Results

### Nodal was highly expressed in CRC

**Fig. 1:**
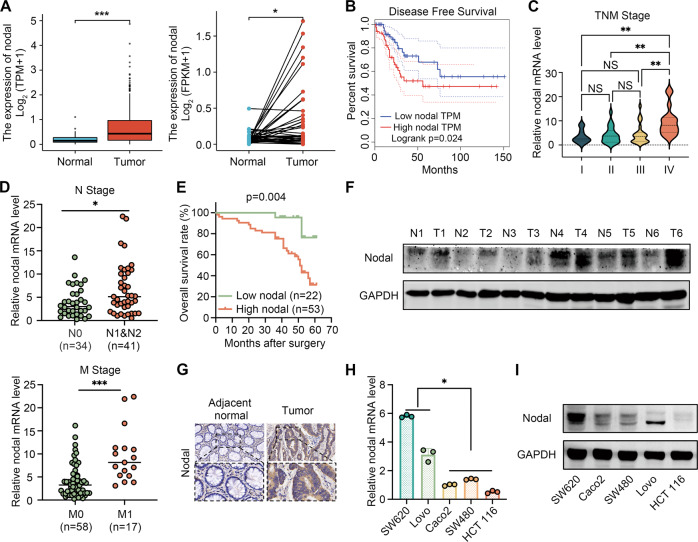
Nodal expression is elevated in CRC, which is associated with the metastasis and poor clinical outcomes of CRC. **A** Expression profile of Nodal in CRC tissues in TCGA cohort. **B** Increased Nodal expression was associated with poor disease-free survival in CRC tissues in TCGA cohort. **C**, **D** Increased Nodal expression was associated with an advanced TNM stage, lymph node metastasis and distant metastasis in 75 CRC tissues obtained from patients. **E** Kaplan–Meier analysis (log-rank test) for the overall survival of 75 patients with CRC in the high- and low-Nodal-expression groups stratified based on the median Nodal expression. **F** Western blotting was performed to evaluate the protein expression of Nodal in six pairs of CRC tissues. **G** Representative images of immunohistochemical (IHC) staining for Nodal in CRC tissues and their adjacent normal tissues (magnification, 200×). **H**, **I** qRT-PCR and western blotting were performed to examine the mRNA and protein expression of Nodal, respectively, in several CRC cell lines. (metastatic cell lines: SW620 and Lovo; non-metastatic cell lines: Caco2, SW480 and HCT116). Data are expressed as mean ± SD of three independent experiments (^∗^*P* < 0.05, ^∗∗^*P* < 0.01, and ^∗∗∗^*P* < 0.001).

The Nodal expression pattern in various types of cancers in The Cancer Genome Atlas (TCGA) database was examined to determine the effects of Nodal on CRC. Fig. S1A illustrates that Nodal expression was higher in cholangiocarcinoma, glioblastoma multiforme, colon cancer, kidney renal clear cell carcinoma, and rectum carcinoma than in the corresponding adjacent normal tissues (ANTs). In addition, it was not only significantly increased in CRC tissues (Fig. 1A) but also closely associated with CRC distant and lymph node metastases (Fig. S1B). A shorter disease-free survival was observed in patients with high Nodal expression (Fig. 1B). Nodal’s mRNA expression was examined in CRC tissues and the corresponding ANTs (*n* = 75) via qRT-PCR to verify these results. It was observed that Nodal expression was significantly up-regulated in CRC (Fig. S1C). In addition, an association was observed between Nodal expression and various tumour progression features, including the tumour size and the advanced tumour, node, and metastasis stage. However, Nodal expression was not linked to clinicopathological parameters, including sex, age, tumour location, and tumour differentiation (Fig. 1C, D; Table 1). Furthermore, Nodal’s prognostic role in CRC was validated via ROC curve analysis (area under the curve = 0.7696) (Fig. S1D). The ROC curve was used to establish the cutoff value for identifying between high and low levels of Nodal expression. We determined the specificity and sensitivity of the test, as well as the Youden index. In this case, the demarcation point of Nodal expression was located at the maximum value of the Yoden index. High Nodal expression was associated with significantly poor overall survival (Fig. 1E). Subsequently, we selected six pairs of patient samples for analysis. Western blotting (Fig. 1F) and IHC analysis (Fig. 1G) revealed that Nodal’s protein levels were elevated in CRC tissues in contrast with paired ANTs. Furthermore, Nodal expression was examined in CRC cell lines. Nodal’s mRNA and protein levels were elevated in metastatic CRC cell lines (Lovo and SW620) relative to non-metastatic CRC cell lines (Caco2, SW480, and HCT116) (Fig. 1H, I). These results highlight Nodal’s crucial role in CRC progression, particularly in metastasis, and suggest that Nodal up-regulation often indicates a poor prognosis.

### Nodal overexpression promoted the proliferation and invasiveness of CRC cells in vivo and in vitro

**Fig. 2:**
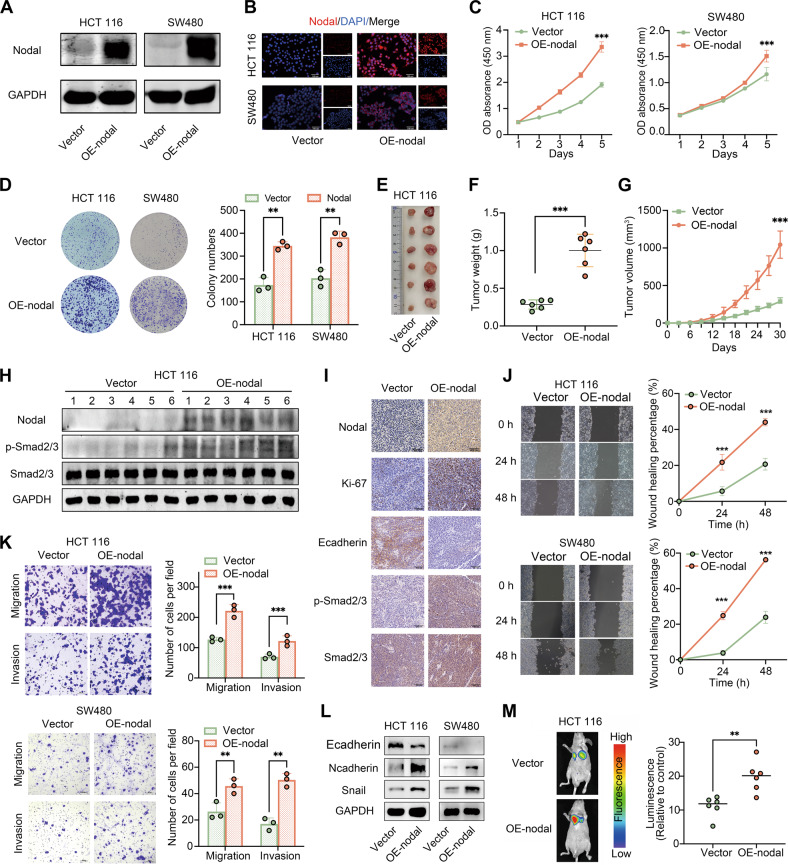
Overexpression of Nodal promoted the proliferation, migration and invasion abilities of CRC cells in vitro and in vivo. **A** Western blotting was performed to examine the protein expression of Nodal in Nodal-overexpressing HCT116 cells and SW480 cells (vector group: cells transfected with an empty vector; oe-Nodal group: cells transfected with the Nodal lentiviral overexpression vector). **B** Representative images of Nodal expression detected in Nodal-overexpressing HCT116 cells and SW480 cells via IF staining. **C**, **D** CCK-8 and colony formation assays validated the increased proliferative ability of HCT116 and SW480 cells after Nodal overexpression. **E** Representative images for the xenograft tumours morphology. **F**, **G** The volume and weight of tumours were evaluated in two groups. **H** Western blotting was performed to examine the expression of Nodal, p-Smad2/3 and Smad2/3 in each xenograft tumor tissue. **I** The Nodal, Ki-67, E-cadherin, p-Smad2/3, and Smad2/3 expressions in xenograft tumour tissues were detected via IHC staining. **J** Wound healing assay revealed that Nodal overexpression increased the migration ability of HCT116 and SW480 cells. **K** Transwell assay validated the increased migration and invasion ability of HCT116 and SW480 cells after Nodal overexpression. **L** Western blotting was performed to examine the E-cadherin, N-cadherin and Snail expressions of HCT116 and SW480 cells in the vector/oe-Nodal group. **M** Representative fluorescence images of lung metastasis in mice and the relative luminescence intensity. (^∗∗^*P* < 0.01 and ^∗∗∗^*P* < 0.001).

The stable models of Nodal-overexpressing HCT116 cells and SW480 cells were established through lentiviral transfection. Nodal’s mRNA and protein levels were substantially elevated in these cells (Fig. 2A; S2A). This was consistent with the IF analysis findings (Fig. 2B). Furthermore, we conducted CCK-8 and colony formation assays to examine Nodal’s role in vitro. The results revealed that Nodal overexpression promoted HCT116 and SW480 cell proliferation (Fig. 2C, D). Following this, a tumour xenograft model was established to examine the influence of Nodal on tumour growth in vivo (Fig. 2E). The model indicated that Nodal overexpression increased the tumour volume and weight after 30 days (Fig. 2F, G), which was in line with the in vitro experiment findings. Furthermore, Ki-67, Nodal, E-cadherin, and Smad2/3 expressions and their phosphorylation levels were identified via Western blotting and IHC analysis in xenograft tumour models (Fig. 2H, I). Wound healing (Fig. 2J) and Transwell (Fig. 2K) assays indicated that Nodal promoted HCT116 and SW480 migration and invasion. The Western blotting results verified that Nodal overexpression induced EMT activation by down-regulating E-cadherin and up-regulating N-cadherin and snail (Fig. 2L). IF analysis revealed consistent findings, indicating that the E-cadherin expression level was considerably lowered and the vimentin level was elevated in oe-Nodal-HCT116 cells (Fig. S2B). Furthermore, Luciferase-labelled HCT116 cells were used to establish a model of lung metastasis to evaluate the pro-metastatic ability of cells in vivo. Bioluminescent imaging indicated that Nodal significantly increased lung metastasis (Fig. 2M). These results indicate that Nodal promotes CRC cell growth and metastasis in vivo and in vitro.

### Knockdown of Nodal inhibited the proliferation and invasiveness of CRC cells in vitro

SiRNA transfection successfully inhibited Nodal in SW620 and Lovo cell lines (Fig. S3A–C), and the first siRNA was selected for the subsequent functional analysis. According to CCK-8 and colony formation assays, Nodal silencing inhibited Lovo and SW620 cell growth (Fig. S3D, E). Wound healing and Transwell assays demonstrated that Nodal silencing lowered SW620 and Lovo cell migration and invasion (Fig. S3F–G). According to IF analysis, most Lovo cells with Nodal silencing demonstrated an elevation in E-cadherin levels and a decrease in vimentin levels (Fig. S3H). Consistently, western blotting revealed that EMT was inhibited via Nodal silencing (Fig. S3I).

### Nodal required SCD1 to promote tumour progression in CRC

**Fig. 3:**
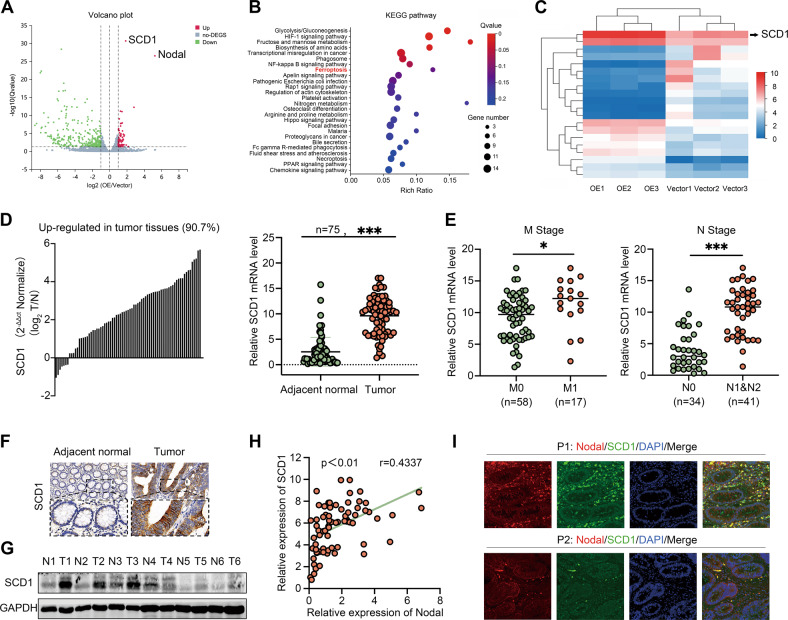
SCD1 may be the potential downstream gene of Nodal. RNA-sequencing was performed using HCT116 cells stably overexpressing Nodal and control cells. The experiment was repeated thrice for each cell type. **A** Volcano map demonstrating the distribution of DEGs in HCT116 cells in the vector/oe-Nodal group. **B** Kyoto Encyclopaedia of Genes and Genomes (KEGG) enrichment analysis showed the signalling pathways that were most enriched by significantly differentially expressed genes. **C** Heatmap demonstrating the relative protein abundance. **D** The mRNA expression of SCD1 in 75 CRC tissues and their adjacent normal tissues was quantified via a qRT-PCR. **E** Correlation between SCD1 expression and the metastasis and node stages in the collected CRC samples. **F** Representative images of IHC staining for SCD1 in CRC tissues and their adjacent normal tissues (magnification, × 200). **G** Western blotting was performed to examine the protein expression of SCD1 in six pairs of CRC tissues. **H** Pearson correlation analysis of the correlation between SCD1 and Nodal expression in the collected CRC samples. The relative expression level was calculated by using the 2^-△△CT^ method. **I** Dual IF assay showed the expression and distribution of the SCD1 (green) and Nodal (red) in two CRC tissues (magnification, 200×). (^∗^*P* < 0.05 and ^∗∗∗^*P* < 0.001).

RNA-Seq was performed to evaluate gene expression in vector-HCT116 cells and oe-Nodal-HCT116 cells to examine the mechanisms underlying the oncogenic effects of Nodal on CRC cells, and DEGs were visualised on a volcano plot (Fig. 3A). The KEGG pathway analysis revealed the top 26 biological and pathological pathways most affected by Nodal overexpression (Fig. 3B). Genes that were most affected by Nodal overexpression were demonstrated using a heatmap. These genes included SCD1, CAV1, ANKRD37 and ANGPTL4 (Fig. 3C). Among these DEGs, SCD1 was one of the most differentially up-regulated genes. Therefore, it was further analysed. TCGA data revealed that SCD1 expression increased in most malignant tumours, including CRC (Fig. S4A, B), and an association was observed between high SCD1 expression and lymph node metastasis and poor survival (Fig. S4C, D). Furthermore, SCD1 expression was examined in 75 pairs of CRC specimens, and an elevated mRNA expression level of SCD1 was discovered in most CRC tissues than in the ANTs (Fig. 3D). Consistent with the database findings, SCD1’s high mRNA expression was also associated with distant/lymphatic metastasis (Fig. 3E). Additionally, IHC staining and Western blotting revealed a higher SCD1 protein expression in CRC tissues than in ANTs (Fig. 3F, G). Following this, SCD1’s effects on proliferation, migration, and invasion were examined by silencing SCD1 in Lovo and SW620 cells using CCK-8 assays, colony formation assays, IF analysis, and Western blotting (Fig. S5A–F). According to the results, SCD1 played a tumour-promoting role in CRC cells. Notably, SCD1 was positively linked to Nodal expressions in both our clinical CRC specimens (*r* = 0.43, *P* < 0.01, Fig. 3H) and TCGA data (Fig. S4E). IF analysis revealed that SCD1 was co-located with Nodal in a similar spatial area in CRC tissues (Fig. 3I). We explored the link between Nodal and SCD1 in CRC progression owing to the correlation between Nodal and SCD1. Subsequently, we performed the rescue assays. It revealed that SCD1 overexpression significantly promoted the attenuated proliferative, migratory, and invasive capacities of Lovo cells triggered by Nodal silencing (Fig. S6A–D), while SCD1 knockdown decreased proliferation, migration, and invasion in Nodal overexpression HCT116 cells (Fig. S6E–H). The significance of SCD1 for Nodal to facilitate CRC progression and metastasis is highlighted by these results.

### Nodal-regulated lipid peroxidation and ferroptosis

**Fig. 4:**
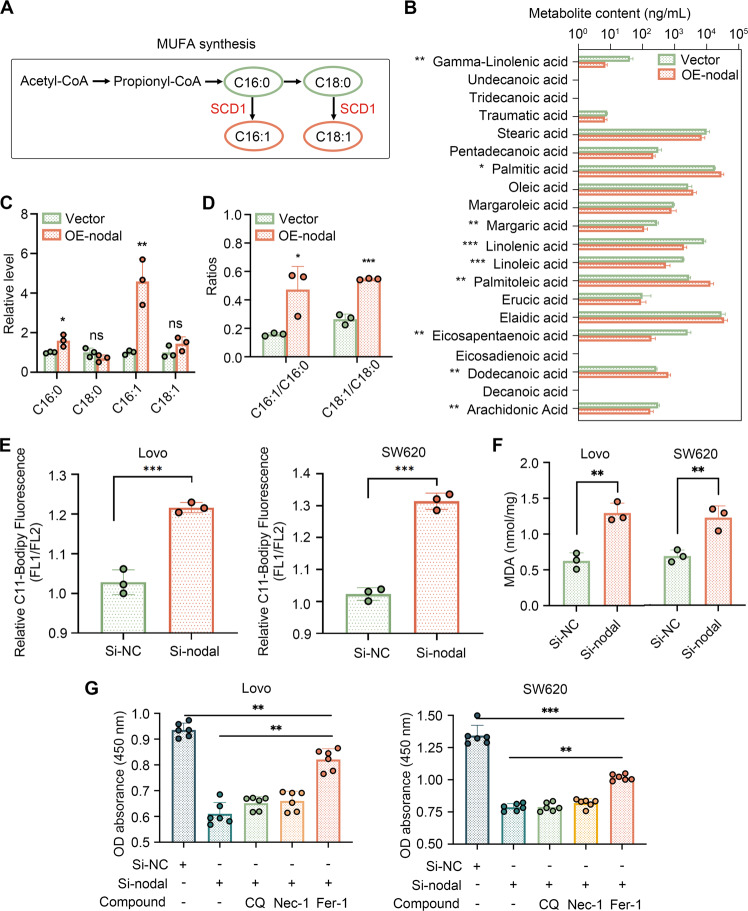
Nodal regulates fatty acid desaturation and lipid peroxidation. **A** Schematic diagram of the synthesis of monounsaturated fatty acids (MUFAs). Monosaturated fatty acids: stearic acid (C18:0) and palmitic acid (C16:0); MUFAs: oleic acid (C18:1) and palmitoleic acid (C16:1). **B** The content of various fatty acids in the vector and oe-Nodal groups. **C** The relative level of the four main fatty acids in oe-Nodal group compared with the vector group. **D** Fatty acid desaturation levels were evaluated based on the ratio of MUFAs to the corresponding monosaturated fatty acids. **E** Flow cytometry was performed to analyse lipid peroxidation in Lovo and SW620 cells stained with BODIPY™ C11 after Nodal knockdown. Fluorescence channel (FL)1 channel (excitation, 488 nm) was used to measure the oxidised lipid, whereas FL2 channel (excitation, 561 nm) was used to measure the unoxidised lipid. The proportion of lipid peroxidation cells was quantified according to the fluorescence intensity ratio of FL1 to FL2. **F** Malondialdehyde (MDA) levels were detected in Lovo and SW620 cells after Nodal knockdown. **G** Cell viability was measured via CCK8 assay after cells in the si-Nodal group were treated with several cell death inhibitors for 48 h. Cell death inhibitors: ferrostatin-1 (Fer-1, a ferroptosis inhibitor, 1 μM), chloroquine (CQ, an autophagy inhibitor, 10 μM), and necrostatin-1 (Nec-1, a necroptosis inhibitor, 10 μM). (^∗^*P* < 0.05, ^∗∗^*P* < 0.01 and ^∗∗∗^*P* < 0.001).

According to the previous KEGG pathway analysis, Nodal might be involved in ferroptosis, and SCD1 is a vital ferroptosis regulator. This illustrates that ferroptosis performs an integral function in accounting for the Nodal-mediated carcinogenic effect. SCD1 catalyses the desaturation of monosaturated fatty acids (primarily stearic acid [C18:0] and palmitic acid [C16:0]) into their corresponding MUFAs (oleic acid [C18:1] and palmitoleic acid [C16:1]) [26] (Fig. 4A), which are indispensable for preventing lipid peroxidation and ferroptosis. We attempted to determine whether Nodal regulated the ferroptosis in a manner similar to SCD1 because SCD1 was the crucial downstream of Nodal in CRC. To confirm this, UPLC-MS/MS was performed to compare changes in the FA profile of oe-Nodal-HCT116 cells and vector-HCT116 cells (Fig. 4B). We examined the relative expression of four FAs associated with the enzymatic activity of SCD1. C16:0 and C16:1 contents were significantly higher in oe-Nodal-HCT116 cells (Fig. 4C). However, the desaturation level reflected by C18:1/C18:0 and C16:1/C16:0 was remarkably elevated following Nodal overexpression (Fig. 4D). The findings indicate that Nodal significantly influences FA metabolism and saturation. Then, an oxidisable lipid ROS probe (C11-BODIPY^581/591^) was used to measure the degree of lipid peroxidation. Additionally, the lipid peroxidation level was reflected by the ratio of green (oxidised lipid) fluorescence intensity to red (non-oxidised lipid) fluorescence intensity via flow cytometry, and lipid peroxide accumulation in CRC cells with Nodal down-regulation was confirmed (Fig. 4E). Similarly, MDA production (a lipid peroxidation product) increased after Nodal silencing (Fig. 4F). These results demonstrate Nodal’s crucial role in lipid peroxidation. Furthermore, we examined whether Nodal silencing lowered cell growth by inducing ferroptosis. It was found that Fer-1 (a ferroptosis inhibitor) reversed the decrease in cell viability caused by Nodal silencing in Lovo and SW620 cells. However, this effect was not brought about by CQ (an autophagy inhibitor) and Nec-1 (a necroptosis inhibitor) (Fig. 4G). This finding illustrated that the decreased cell activity triggered by Nodal silencing was partly mediated by ferroptosis-induced cell death.

### Nodal affected RSL3‐triggered ferroptosis through SCD1

**Fig. 5:**
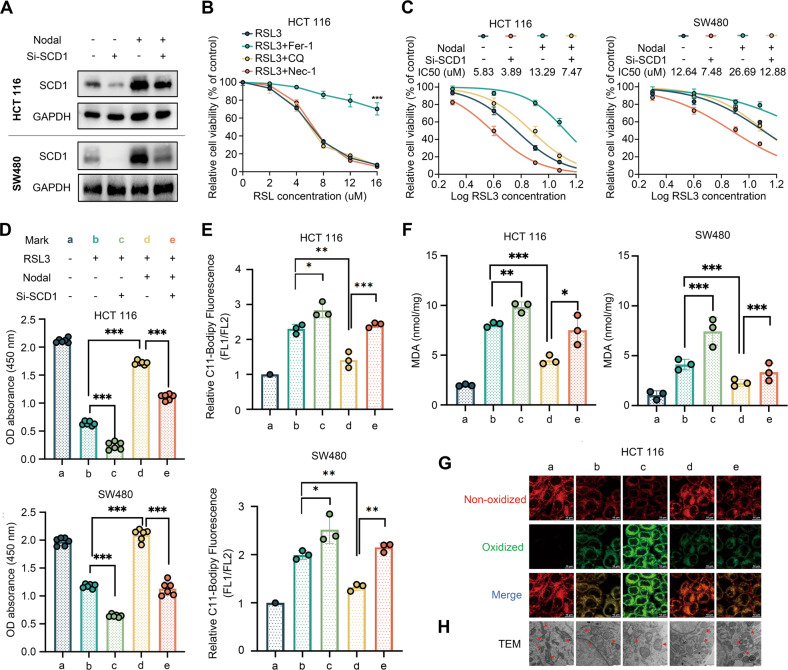
Nodal regulated the sensitivity to RSL3-induced ferroptosis via SCD1. **A** Western blotting of SCD1 in HCT116 and SW480 cells in the vector and oe-Nodal groups with or without si-SCD1 transfection. **B** Relative cell viability of HCT116 cells treated with different concentrations of RSL3 (a ferroptosis inducer) for 48 h with or without several cell death inhibitors. **C** Relative cell viability of HCT116 and SW480 cells in the vector and oe-Nodal groups with or without si-SCD1 transfection. The cells were treated with different concentrations of RSL3 for 48 h. **D** Cell viability was measured via CCK8 assay in HCT116 and SW480 cells in the vector and oe-Nodal groups with or without si-SCD1 transfection. HCT 116 cells and SW480 cells were treated with 8 μM and 12 μM RSL3 for 48 h, respectively. **E** BODIPY™ C11 staining analysis of lipid peroxidation via flow cytometry in Nodal-overexpressing HCT116 and SW480 cells with or without si-SCD1 transfection. **F** MDA levels in HCT116 and SW480 cells in the vector and oe-Nodal groups with or without si-SCD1 transfection. **G** Representative confocal laser scanning microscopic images of lipid peroxidation in HCT116 cells (green: oxidised lipids; red: non-oxidised lipids). **H** Representative transmission electron microscopic images of morphological changes in mitochondria of HCT116 cells (red arrows: mitochondria). (^∗^*P* < 0.05, ^∗∗^*P* < 0.01 and ^∗∗∗^*P* < 0.001).

oe-Nodal-HCT116/SW480 cells and vector-HCT116/SW480 cells were transfected with SCD1 siRNA to knock down endogenous SCD1 expression to verify SCD1’s involvement in Nodal-regulated ferroptosis, and the transfection efficiency was examined (Fig. 5A). Subsequently, the cells were treated with RSL3. We confirmed that as a ferroptosis inducer, RSL3 dosage dependently triggered cell death and only Fer-1 reversed these effects (Fig. 5B). Nodal overexpression reduced HCT116 and SW480 cell sensitivity to RSL3, whereas SCD1 inhibition restored the sensitivity to a certain extent. The half-maximal inhibitory concentration (IC_50_) values of the several groups were as follows: vector-HCT116, 5.83 μM; Nodal-HCT116, 13.29 μM, (vector+si-SCD1)-HCT116, 3.89 μM; (Nodal+si-SCD1)-HCT116, 7.472 μM; vector-SW480, 12.64 μM; Nodal-SW480, 26.69 μM; (vector+si-SCD1)-SW480, 7.48 μM; (Nodal+si-SCD1)-SW480, 12.88 μM (Fig. 5C). In addition, Nodal overexpression decreased RSL3-induced cell death and RSL3-induced lipid peroxide and MDA generation in HCT116 and SW480 cells, which was reversed after SCD1 knockdown (Fig. 5D–F). Consistently, confocal microscopy validated that Nodal overexpression lowered oxidised lipid levels in HCT116 cells treated with RSL3 but not in cells with SCD1 silencing (Fig. 5G). Furthermore, TEM revealed that RSL3 significantly impacted the mitochondrial morphology, resulting in mitochondrial swelling, a decrease in the number of mitochondrial cristae or their complete absence, and rupture of the outer membrane of the mitochondria, which are the characteristics of ferroptosis. oe-Nodal-HCT116 cells exhibited the least mitochondrial damage compared with other cells (Fig. 5H). These results indicate that Nodal regulates the sensitivity to RSL3-induced ferroptosis via SCD1.

### Nodal promoted SCD1 transcription via the Smad2/3 pathway

**Fig. 6:**
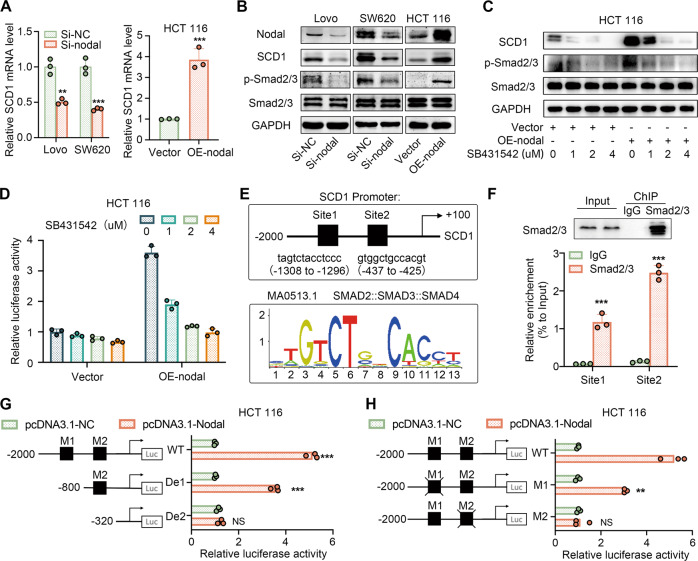
Nodal regulated SCD1 transcription through Smad2/3. **A** qRT-PCR analysis of SCD1 mRNA after Nodal down-regulation and up-regulation. **B** Western blot analysis of the protein expression of Nodal, SCD1, Smad2/3 and p-Smad2/3 after Nodal down-regulation and up-regulation. **C** Western blot analysis of the protein expression of Nodal, SCD1, Smad2/3 and p-Smad2/3 in vector and oe-Nodal HCT116 cells treated with different concentrations of SB431542. **D** A dual luciferase reporter assay system was used to measure the activity of SCD1 promoter in HCT116 cells in the vector and oe-Nodal groups treated with different concentrations of SB431542. **E** The sequence logo of the potential smad2::smad3::smad4 binding site predicted using the JASPAR database and schematic diagram of the predicted binding sites in the SCD1 promoter sequence (site 1: from –1308 to –1296 bp, site 2: from –437 to –425 bp). **F** ChIP assay was performed using a Smad2/3-specific antibody and an IgG control antibody. Western blotting was performed to analyse bait-specific bands in cell lysates and ChIP complexes to evaluate the success of ChIP experiment. qPCR was performed to analyse the expression of immunoprecipitated DNA. **G**, **H** HCT116 cells were co-transfected with different pGL3-SCD1 promoter plasmids and pcDNA3.1-NC/pcDNA3.1-Nodal, and the luciferase activity of SCD1 was examined. Note: WT, full-length wild-type plasmid (–2000 to +100 bp); De1, truncation plasmid (–800 to +100 bp); De2, truncation plasmid (−320 to +100 bp); Mut1, plasmid in which the first binding site was deleted; Mut2, plasmid in which the second binding site was deleted). (^∗∗^*P* < 0.01 and ^∗∗∗^*P* < 0.001).

We aimed to identify the mechanism by which Nodal and SCD1 were associated with ferroptosis and tumour progression. As a member of the TGF-β superfamily, Nodal primarily binds to its membrane receptors to transmit signals to Smad2 and/or Smad3, thereby regulating related gene transcription [27]. Therefore, we examined the Nodal–Smad2/3 signalling pathway’s role in regulating SCD1 transcription. qRT-PCR and Western blotting revealed that Nodal down-regulation reduced Smad2/3 phosphorylation and SCD1’s mRNA and protein expression, whereas Nodal up-regulation had the opposite effect (Fig. 6A, B). This finding revealed Nodal’s regulatory effects on SCD1. Vector-HCT116 and oe-Nodal-HCT116 cells were treated with SB431542 (Smad2/3 pathway inhibitor), respectively, to verify the role of Smad2/3 in regulating Nodal’s effect. The results revealed that Smad2/3 phosphorylation and SCD1 expression were concentration-dependently suppressed by SB431542 (Fig. 6C). Dual luciferase reporter assay suggested that SCD1 promoter’s transcriptional activity increased in Nodal-overexpressing cells but gradually decreased with an increase in SB431542 concentration (Fig. 6D). Furthermore, two predicted binding domains for Smad2/3 in the SCD1 promoter were found using the JASPAR database (Fig. 6E), and the direct binding between Smad2/3 and the two predicted sites was detected by ChIP assay. The Smad2/3 protein served as a bait, and protein bands corresponding to the expected molecular mass were detected in cell lysates and experimental ChIP complexes but not in the IgG group, indicating the success of the ChIP experiment. Subsequently, qPCR revealed that the two predicted binding sites were enriched after immunoprecipitation with anti-Smad2/3 antibodies (Fig. 6F), indicating a direct interaction between Smad2/3 and the SCD1 promoter at the two sites. Luciferase reporter assay was performed on 293T and HCT116 cells transfected with pGL3-SCD1 promoter plasmids to determine the functionality of the two binding sites, and the findings suggested that pGL3-SCD1 (−2000 bp to −320 bp) was required for SCD1’s transcriptional activity (Figs. 6G; S7A). Luciferase gene expression was regulated by two pGL3-SCD1 promoter plasmids that deleted the Smad2/3 binding sites (M1 and M2) and a wild-type promoter plasmid (control). The results revealed that Nodal-induced SCD1 promoter activation was retained to some extent after deleting the first binding site but was almost eliminated after deleting the second binding site (Figs. 6H; S7B). Despite both sites having the potential to bind to Smad2/3, only the second site contributed to SCD1 transcription. These findings indicate that Nodal can promote SCD1 transcription by activating the Smad2/3 signalling pathway and facilitating binding between Smad2/3 and the SCD1 promoter.

### BSA-NPs encapsulating Nodal siRNA indicated a good anti-tumour efficacy

**Fig. 7:**
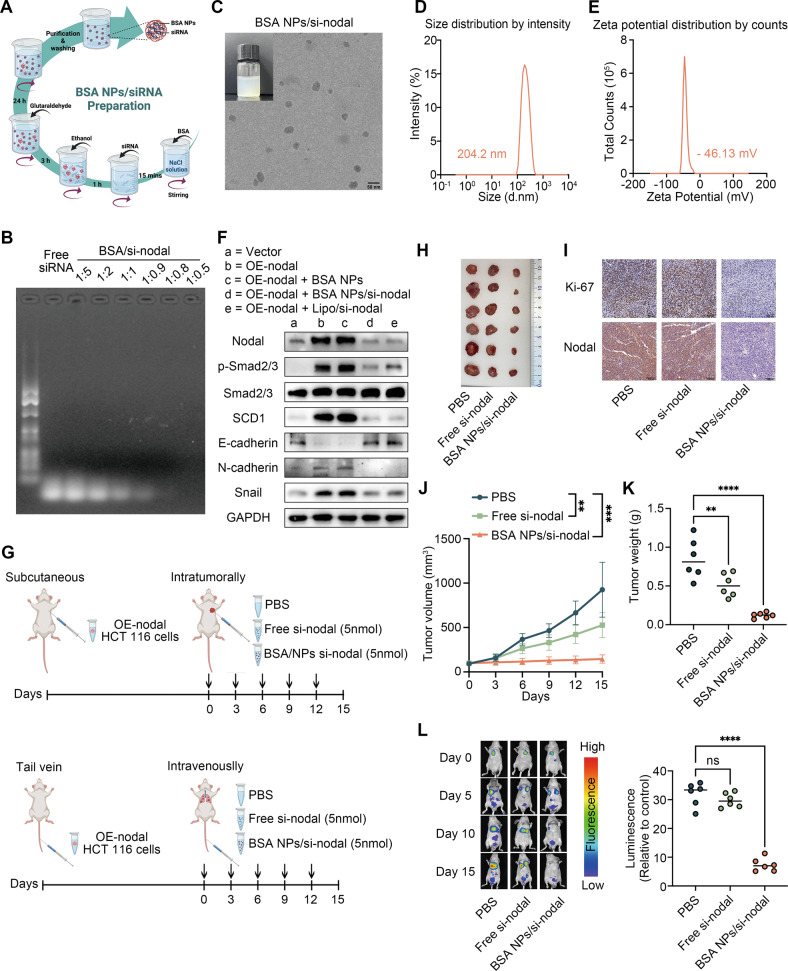
BSA-NP/si-Nodal nanocomplexes inhibit the proliferation and metastasis of CRC. **A** Schematic diagram for the preparation of BSA-NP/si-Nodal nanocomplexes. **B** Agarose gel retardation assay was performed to evaluate the encapsulation efficiency of BSA-NPs. **C** Transmission electron microscopic images of BSA-NP/si-Nodal nanocomplexes. **D**, **E** The hydrodynamic particle size and zeta potential distribution of BSA-NP/si-Nodal complexes were measured via dynamic light scattering. **F** Western blot analysis of Nodal, SCD1, Smad2/3, p-Smad2/3, E-cadherin, N-cadherin and snail expressions in cells with different treatments. **G** Schematic diagram of in vivo experiments in tumour-bearing mice models. **H**–**J** Tumour images, tumour volume and tumour weight in mice. **K** IHC analysis of the Ki67 and Nodal expressions. **L** Fluorescence images and fluorescence intensity analysis of nude mice with lung metastasis (^∗∗^*P* < 0.01 and ^∗∗∗^*P* < 0.001).

As Nodal up-regulation is crucial to CRC progression, it was silenced using the specific siRNA for cancer therapy. BSA-NPs were prepared via a desolvation-crosslinking method to encapsulate and deliver si-Nodal to CRC cells (Fig. 7A). We prepared six nano complexes with different BSA to si-Nodal weight ratios (mg/μg). According to the agarose gel retardation assay, the highest loading efficiency was obtained when the ratio was 1:0.8 (Fig. 7B), which was selected for further BSA-NP/si-Nodal complex preparations. A bluish opalescent appearance was exhibited by the synthesised BSA-NP/si-Nodal complexes in the solution, and a uniformly distributed spherical morphology was observed on TEM (Fig. 7C). Dynamic light scattering revealed that the BSA-NP/si-Nodal complex had a hydrodynamic diameter and zeta potential of 204.2 nm and –46.13 mV, respectively (Fig. 7D, E). Subsequently, in vitro functional experiments in HCT116 cells revealed that BSA-NP/si-Nodal treatment exerted the same anti-proliferative, anti-migratory, and anti-invasive effects on Nodal-HCT116 cells as on the positive control cells (Lipo/si-Nodal) compared with BSA-NP treatments (Fig. S8A–D). Western blotting indicated that BSA-NP/si-Nodal significantly inhibited the downstream Nodal signalling pathway activation (Fig. 7F). Furthermore, BSA-NP/si-Nodal nano complex’s inhibitory effects on tumour growth and metastasis were examined in tumour-bearing mice models (Fig. 7G). A remarkable reduction was observed in the tumour volume and weight in the BSA-NP/si-Nodal-treated group (Fig. 7H), and IHC staining was performed for further detection of the related molecule (Fig. 7I). In addition, bioluminescent imaging revealed that BSA-NP/si-Nodal treatment inhibited lung metastasis in vivo (Fig. 7L). Haematoxylin and eosin staining were performed to assess BSA-NP/si-Nodal’s organ toxicity, and there was no prominent toxic damage to the main organs (kidneys, lungs, spleen, liver, and heart) (Fig. S8E). SCD1 overexpression was performed in BSA-NP/si-Nodal treated cells since Nodal promoted CRC advancement and metastasis by targeting SCD1. According to the biological assays, BSA-NP/si-Nodal’s suppressive effect was mitigated by SCD1 overexpression (Fig. S8F–I). These findings verified the anti-tumour efficacy and biosafety of BSA-NP/si-Nodal suggestive of great potential for CRC therapy.

## Discussion

According to recent studies, the re-emerging Nodal signalling is fundamentally required for human malignancies with an aggressive phenotype, such as breast cancer, prostate cancer, and invasive melanoma [28–30]. Herein, the Nodal expression level was shown to be elevated in CRC tissues, particularly in those with lymph node or distant metastasis. An association was observed between the abnormally high Nodal expression and a severe clinical stage and worse prognosis. These data confirmed the Nodal re-expression as an indicator of cancer aggressiveness. Although no significant association was observed between Nodal expression and the I-III stage, this is not completely consistent with the previous view that Nodal expression was associated with the tumour staging [31, 32]. IV-stage patients still showed a substantial increase in Nodal expression levels. Functionally, Nodal overexpression enhanced the proliferation, migration, and EMT of CRC cells, along with tumour growth in vivo, while Nodal knockdown played the opposite role. Furthermore, good anti-tumour efficacy was demonstrated by BSA-NPs encapsulating Nodal-specific siRNA treatment in vitro and in vivo. These above findings highlight that Nodal signalling is fundamentally required for aggressive and metastatic human malignancies, with promising clinical applicability. Besides, previous studies and our findings (data not shown) suggest that the abnormally high Nodal expression in CRC is possibly induced by hypoxia. The impact of Nodal on the association between tumour and stromal cells in the tumour microenvironment has been addressed [33, 34]. Therefore, further details are required to explore Nodal’s role in a hypoxic microenvironment.

Nodal’s biological role in various cancer types has been expanded in this study. The specific role of Nodal in the malignant evolution of cancers, such as inducing the EMT phenotype, decreasing chemosensitivity, and promoting the self-renewal of cancer stem cells, has been previously described [12, 13, 35–37]. Herein, Nodal served as an antioxidant by protecting cancer cells from ferroptosis and lipid peroxidation damage. A recent study has demonstrated Nodal’s role in preventing ROS accumulation [38]. Besides, Nodal mitigates cerebral ischaemia-reperfusion injury by inhibiting oxidative stress [14]. Studies have reported that metastatic cells in the blood undergo ferroptosis, thereby restricting their survival, and anti-ferroptosis treatment significantly increases metastasis [15, 39]. The higher metabolic demands and ROS load of highly malignant tumour cells are responsible for this. We speculate that Nodal induces aggressive cancer cell resistance to ferroptosis, which is indispensable for the fight against metabolic and oxidative burdens for survival against metastasis.

Herein, RNA-Seq identified SCD1 as the downstream of Nodal. In addition to correlating the expression pattern in CRC tissues, an association was observed between Nodal function and SCD1 expression. SCD1 overexpression restored the decreased CRC cell proliferation and migration caused by Nodal knockdown, while SCD1 inhibition weakened the increased proliferative and migratory abilities of overexpressing Nodal cells. Mechanically, we observed that Nodal promoted SCD1 transcription by activating the Smad2/3 pathway. Reportedly, SCD1 is implicated in the biological behaviour of cancer cell proliferation, migration, and metastasis, maintaining the characteristics of cancer stem cells [40]. Consistent with our results, this provides a basis for evaluating the inhibition of cancer progression and metastasis by SCD1 targets.

However, our study has some limitations. First, although we observed that Nodal overexpression increased the MUFA levels and FA desaturation, the MUFA synthesis enzyme SCD1 was downstream of Nodal. Whether Nodal affected MUFA synthesis through SCD1 requires further study. Second, the specific molecular mechanisms by which Nodal regulated ferroptosis were not determined. Reportedly, MUFAs displace PUFAs from plasma membrane phospholipids, thereby inhibiting lipid ROS accumulation specifically at the plasma membrane [41]. Ovarian cancer cells with SCD1 overexpression show an increase of unsaturated FAs in the membrane, thereby inducing resistance to ferroptosis [21]. Initially, our study demonstrated that Nodal exhibited protection against RSL3-induced ferroptosis by targeting SCD1. But whether Nodal increases MUFA levels through SCD1, followed by replacing PUFAs in the membrane to resist ferroptosis, requires further study. In addition to MUFAs, partial PUFAs, such as arachidonic acid (AA), were also affected by Nodal. Phosphatidylethanolamine and phosphatidylcholine comprising AA are the primary targets for lipid peroxidation [42]. Exogenous AA reportedly enhances the ferroptotic response in RSL3-treated cells [43]. We observed a decrease in the AA content because of Nodal overexpression; however, whether this reduction contributed to Nodal-induced ferroptotic resistance remains unknown. In short, ferroptosis regulation is complicated, and further exploring the link between Nodal, FA metabolism, and ferroptosis will be of interest.

## Conclusion

**Fig. 8:**
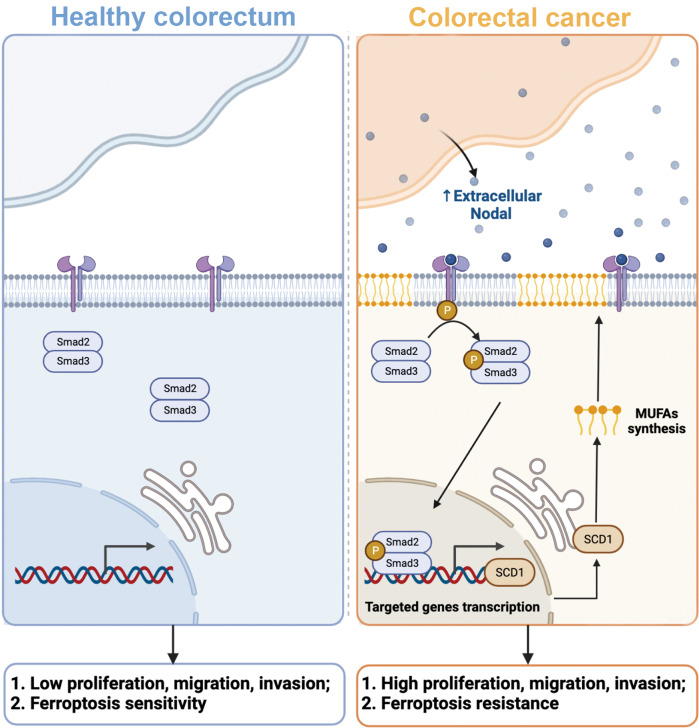
Mechanism of CRC pathogenesis. In the CRC microenvironment, Nodal is abundantly overexpressed and secreted by CRC cells, which subsequently activates the Smad2/3 signalling pathway. Activation of the Smad2/3 signalling pathway increases the expression of SCD1 at the transcriptional level, thereby increasing fatty acid desaturation. This contributes to the resistance of tumour cells to ferroptosis and promotes cell proliferation, migration, and invasion.

Nodal, re-expressed in CRC cells, is an essential driver gene for CRC growth and metastasis that activates Smad2/3–SCD1 signalling (Fig. 8). Advanced delivery carriers should be developed for targeted Nodal silencing therapy and novel therapeutic strategies for CRC using Nodal inhibitors alone or in combination with ferroptosis inhibitors should be developed.

## Supplementary information

Reproducibility checklist

Supplementary figure 1

Supplementary figure 2

Supplementary figure 3

Supplementary figure 4

Supplementary figure 5

Supplementary figure 6

Supplementary figure 7

Supplementary figure 8

Supplementary Information

original western blots

## Data Availability

All data during this study are available from the corresponding author upon reasonable request.
