# Supplementary figures and images for: Nodal promotes colorectal cancer survival and metastasis through regulating SCD1-mediated ferroptosis resistance
Source: Cell Death Dis. 2023 Mar 31;14(3):229. doi: 10.1038/s41419-023-05756-6 (PMC10066180; doi:10.1038/s41419-023-05756-6)

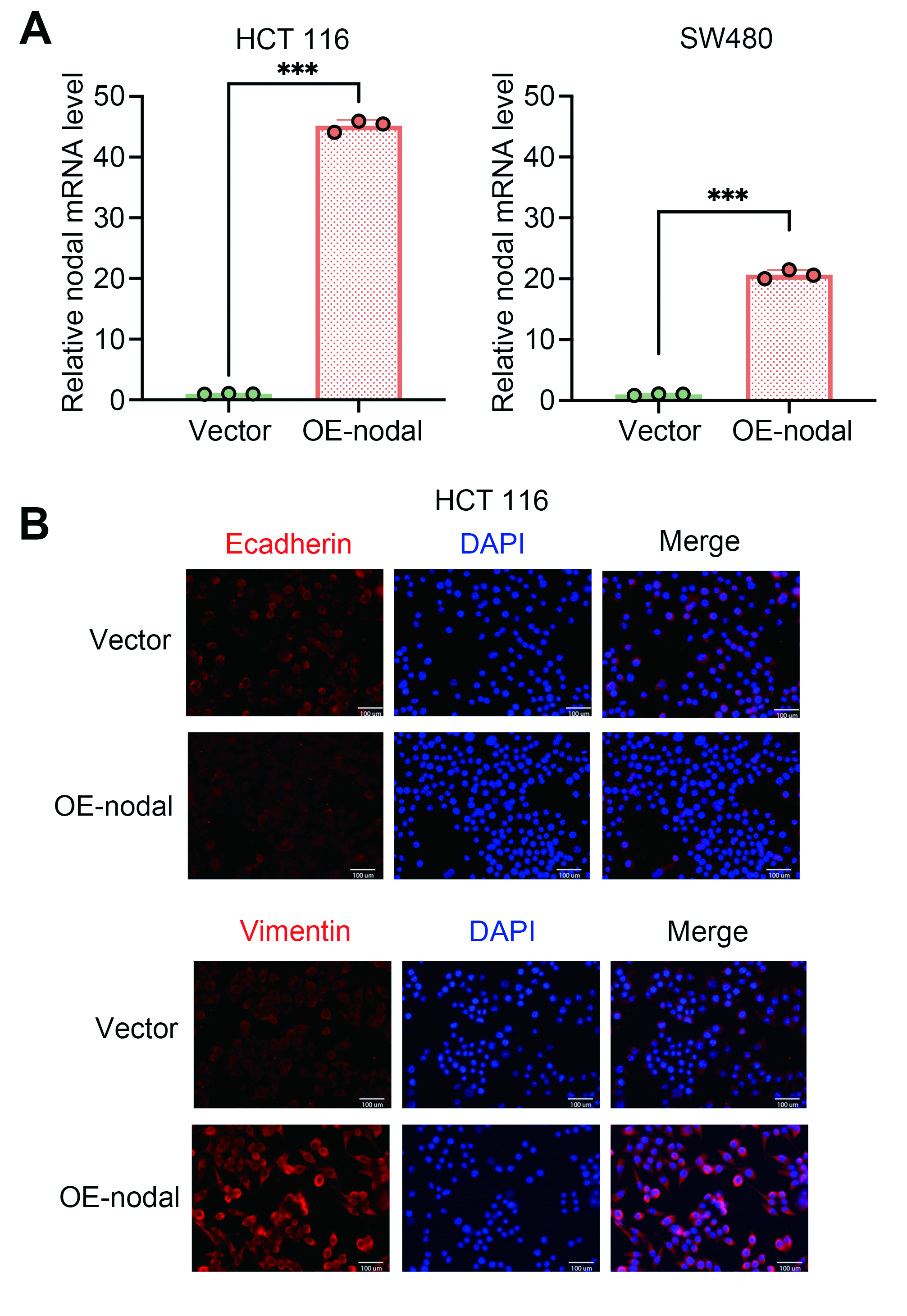

Supplement: Supplementary file 3 — Supplementary figure 2 [file 41419_2023_5756_MOESM3_ESM.tif]

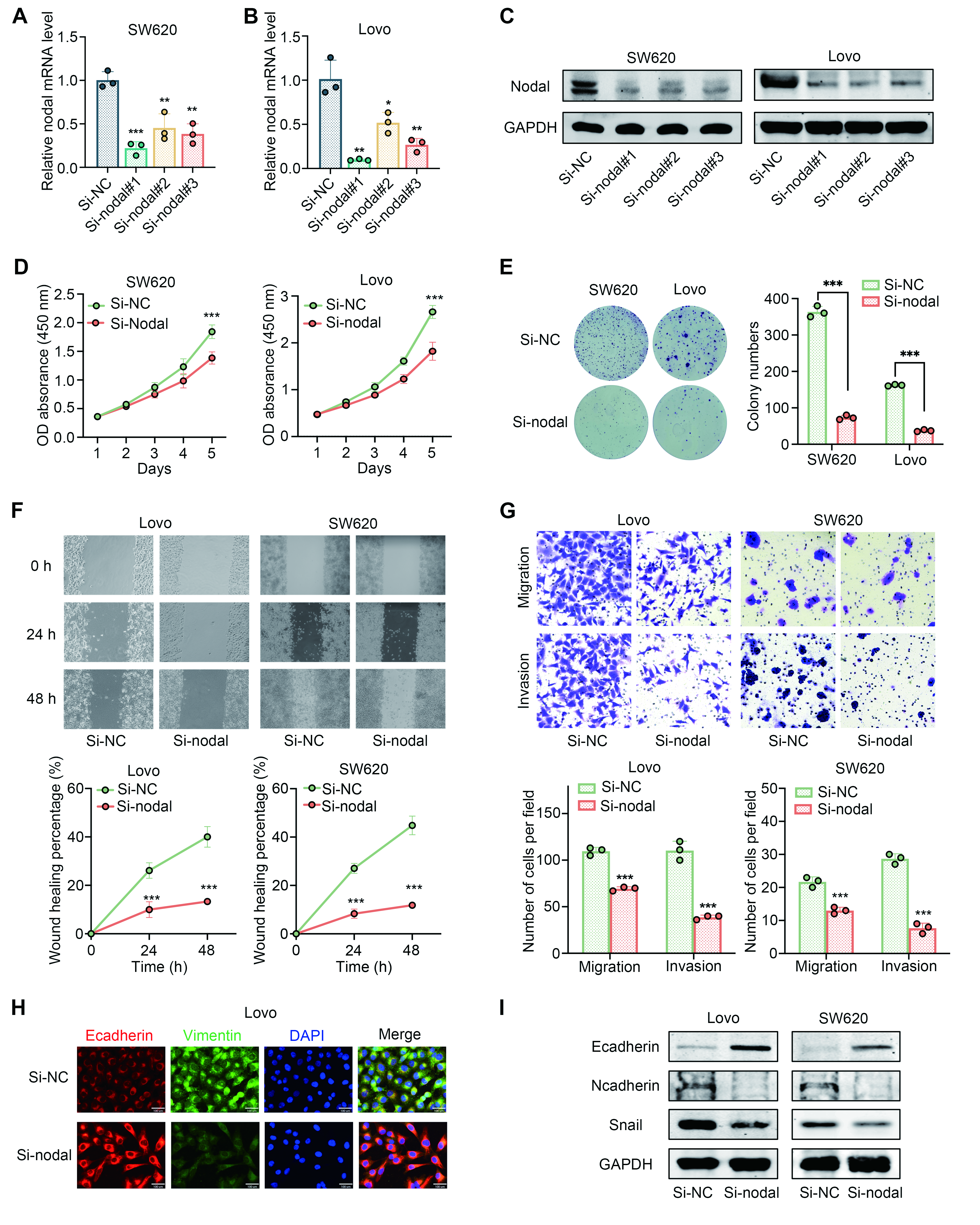

Supplement: Supplementary file 4 — Supplementary figure 3 [file 41419_2023_5756_MOESM4_ESM.tif]

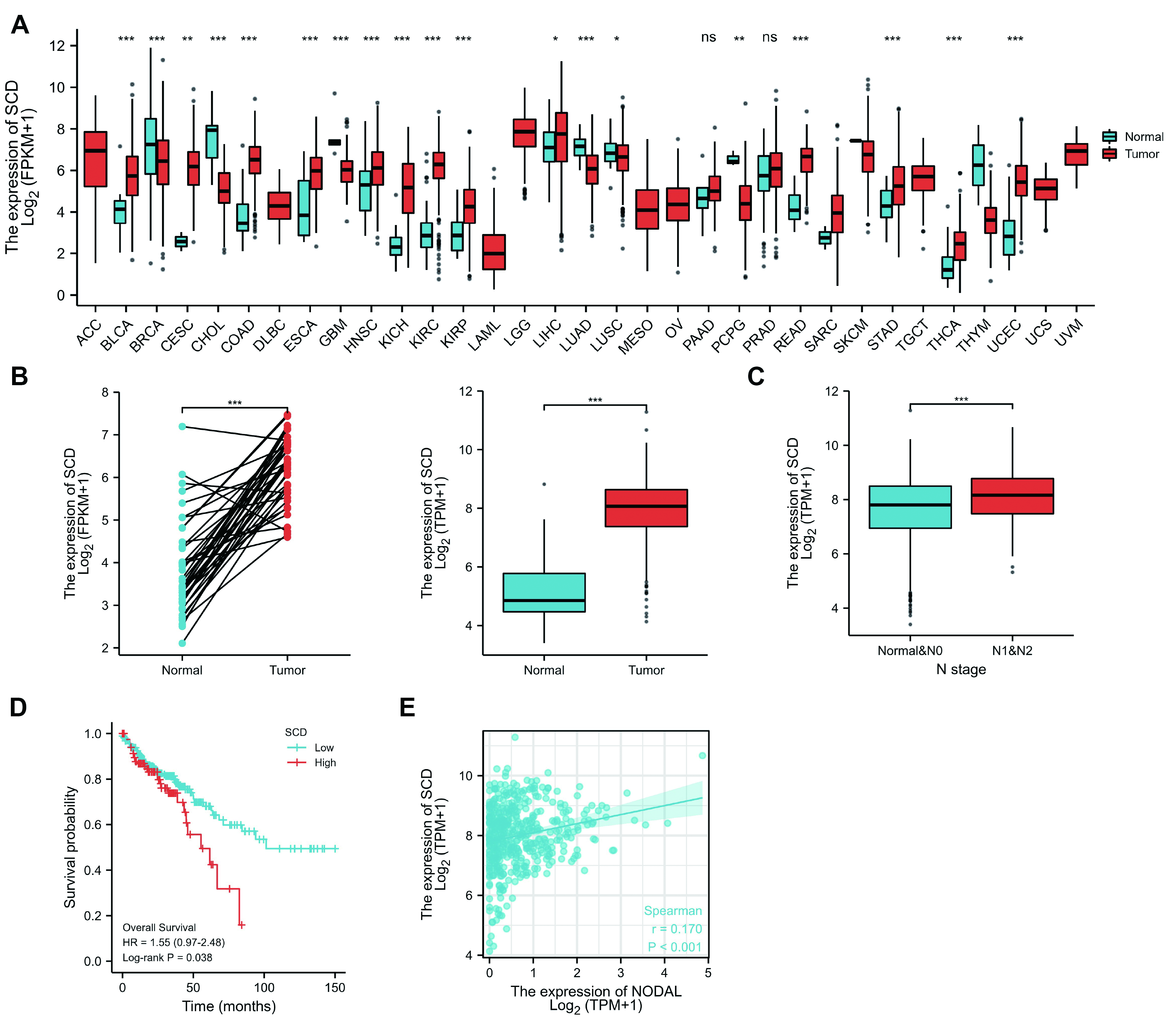

Supplement: Supplementary file 5 — Supplementary figure 4 [file 41419_2023_5756_MOESM5_ESM.tif]

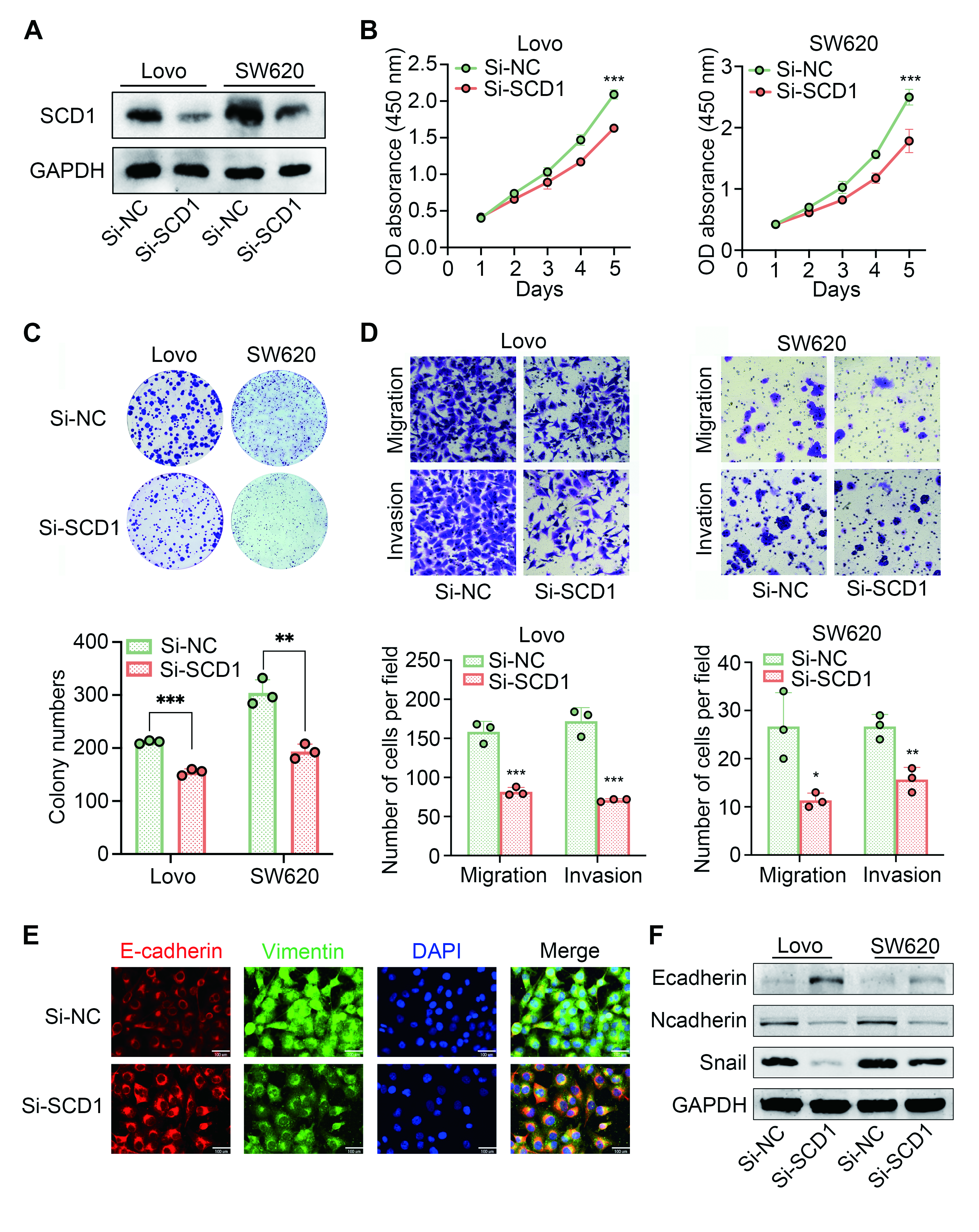

Supplement: Supplementary file 6 — Supplementary figure 5 [file 41419_2023_5756_MOESM6_ESM.tif]

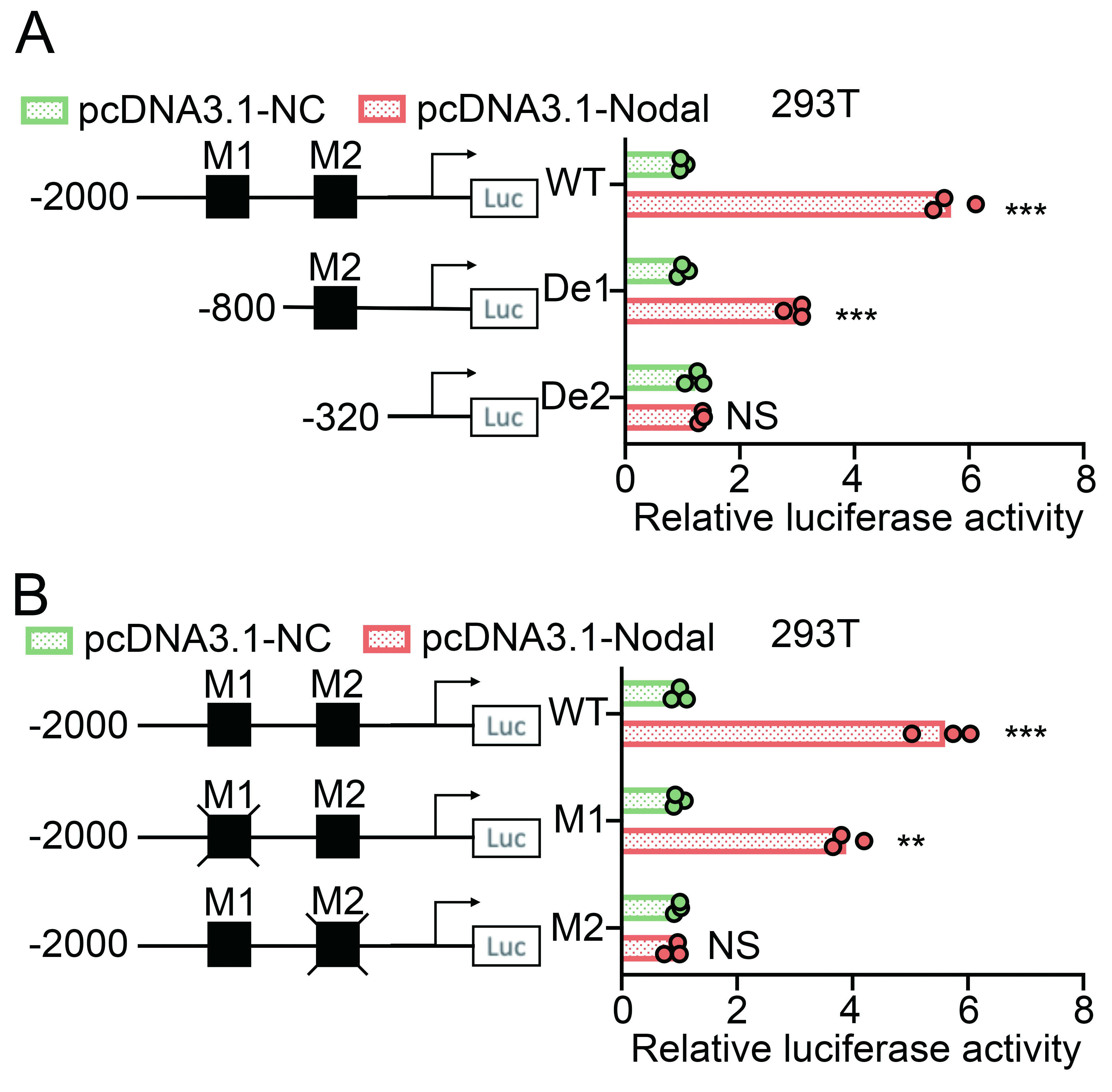

Supplement: Supplementary file 8 — Supplementary figure 7 [file 41419_2023_5756_MOESM8_ESM.tif]

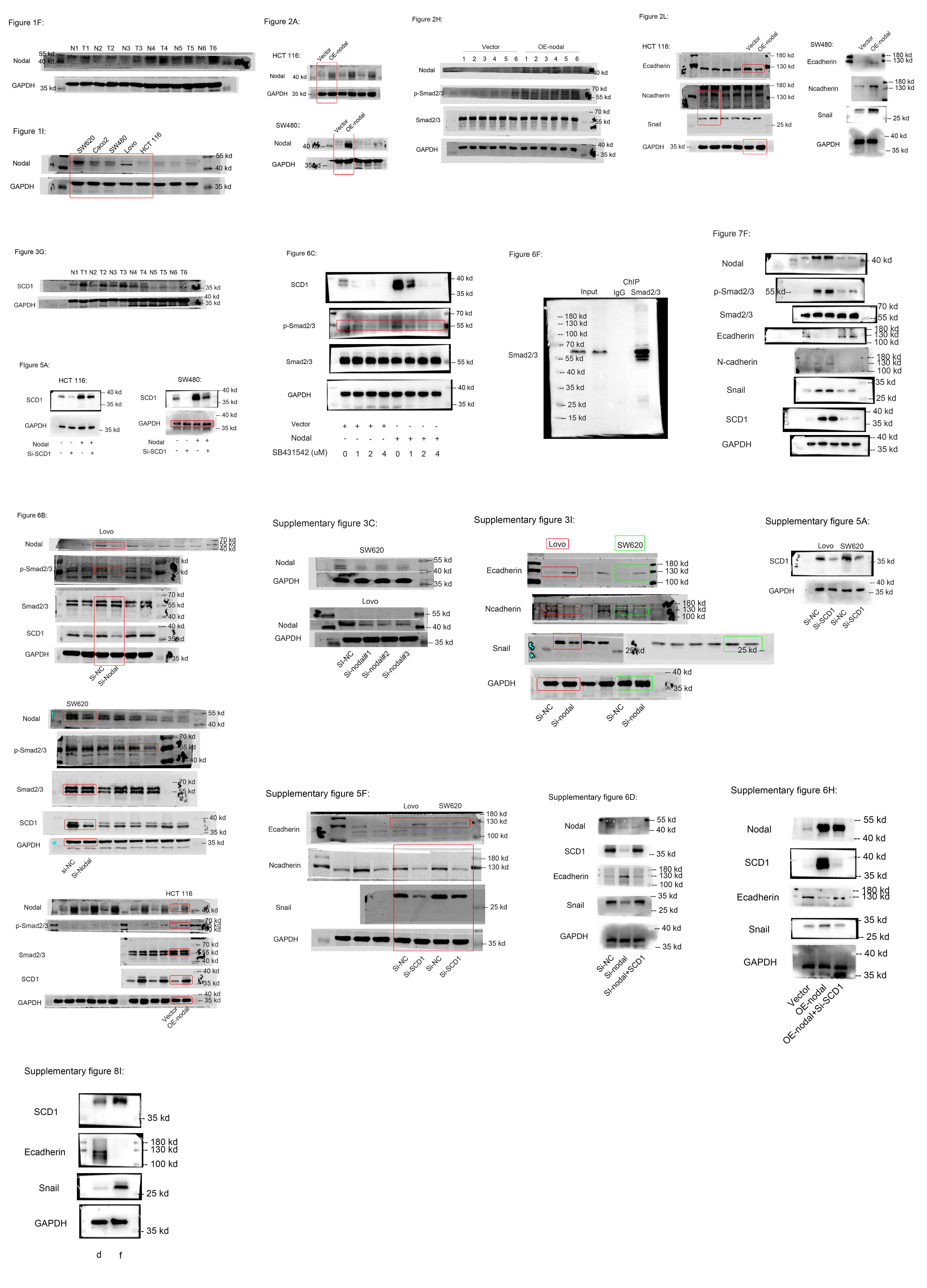

Supplement: Supplementary file 11 — original western blots [file 41419_2023_5756_MOESM11_ESM.jpg]
