# Supplementary material for: Nodal promotes colorectal cancer survival and metastasis through regulating SCD1-mediated ferroptosis resistance
Source: Cell Death Dis. 2023 Mar 31;14(3):229. doi: 10.1038/s41419-023-05756-6 (PMC10066180; doi:10.1038/s41419-023-05756-6)
Supplement: Supplementary file 10 — Supplementary Information [file 41419_2023_5756_MOESM10_ESM.docx]

Supplementary Information for:

# Nodal promotes colorectal cancer survival and metastasis through regulating SCD1-mediated ferroptosis resistance

**Figure legends：**

**Figure S1:**

**Nodal was overexpressed in CRC**

A. Expression profile of Nodal in pan-cancer data extracted from TCGA database.

B. Correlation between Nodal expression and the N and M stages of CRC based on TCGA data.

C. Nodal expression in 75 CRC tissues and the adjacent normal tissues collected from patients was quantified via qRT-PCR.

D. ROC curve for Nodal (area under the curve [AUC] = 0.7696, cut-off = 1.927, 95% confidence interval [CI] = 0.6619–0.8773, Pp＜0.001).

(∗P < 0.05, ∗∗P < 0.01 and ∗∗∗P < 0.001)

**Figure S2:**

**Nodal overexpression promoted EMT**

A. qRT-PCR was performed to examine the mRNA expression of Nodal in Nodal-overexpressing HCT116 and SW480 cells.

B. Representative IF staining images for E-cadherin, N-cadherin and Vimentin (magnification, 100×).

(∗∗∗P < 0.001)

**Figure S3：**

**Nodal silencing reduced the proliferation, migration and invasion abilities of CRC cells in vitro**

A–C. Three small interfering RNAs targeting Nodal (Si-nodal#1, #2 and #3) were designed, and the interference levels of Nodal mRNA and protein after their transfection were detected via qRT-PCR and western blotting, respectively.

D–E. CCK-8 and colony formation assays confirmed the decreased proliferation ability of cells after Nodal knockdown.

F–G. Wound healing and transwell assays confirmed the decreased migration and invasion abilities of cells after Nodal knockdown.

H–I. Immunofluorescence staining and western blotting indicated the reduction in EMT in Lovo cells after Nodal knockdown (magnification, 100×).

(∗∗∗P < 0.001)

**Figure S4：**

**SCD1 was highly expressed in CRC**

A–B. Expression profile of SCD1 in pan-cancer and CRC data extracted from TCGA database.

C–D. Correlation between SCD1 expression and the N stage and survival of patients with CRC based on TCGA data.

E. Correlation between SCD1 and Nodal expression in CRC based on TCGA data.

(∗∗∗P < 0.001).

**Figure S5：**

**SCD1 silencing reduced the proliferation, migration and invasion abilities of CRC cells in vitro**

A. Western blot analysis of SCD1 expression in SW620 and Lovo cells transfected with siRNA targeting SCD1.

B–C. CCK-8 and colony formation assays confirmed the decreased proliferation ability of cells after SCD1 knockdown.

D. Transwell assay confirmed the decreased migration and invasion abilities of cells after SCD1 knockdown.

E–F. IF staining and western blotting indicated the reduction in EMT in Lovo cells after SCD1 knockdown (magnification, 100×).

(∗∗P < 0.01 and ∗∗∗P < 0.001)

**Figure S6：**

**Nodal promoted the proliferation, migration and invasion abilities of CRC cells through SCD1**

A-B. CCK-8 and colony formation assays confirmed the SCD1 overexpression rescued the decreased proliferation ability of Lovo cells caused by Nodal knockdown.

C. Transwell assay confirmed the SCD1 overexpression rescued the decreased migrative and invasive ability of Lovo cells caused by Nodal knockdown.

D. Western blot confirmed the SCD1 overexpression rescued the EMT inhibition of Lovo cells caused by Nodal knockdown.

E-F. CCK-8 and colony formation assays confirmed the SCD1 silencing decreased the enhanced proliferation ability of Nodal overexpressing HCT116 cells.

G. Transwell assay confirmed the SCD1 silencing decreased the enhanced migrative and invasive ability of Nodal overexpressing HCT116 cells.

H. Western blot confirmed the SCD1 silencing decreased the EMT promotion of Nodal overexpressing HCT116 cells.

**Figure S7：**

**Nodal regulated the transcriptional activity of SCD1**

HEK 293T cells were co-transfected with different pGL3-SCD1 promoter plasmids and pcDNA3.1-NC/pcDNA3.1-Nodal.

A–B: Luciferase activity of SCD1. Note: WT, full-length wild-type plasmid (–2000 to +100 bp); De1, truncation plasmid (–800 to +100 bp); De2, truncation plasmid (–320 to +100 bp); Mut1, plasmid in which the first binding site was deleted; Mut2, plasmid in which the second binding site was deleted

(∗∗P < 0.01 and ∗∗∗P < 0.001)

**Figure S8：**

**BSA-NP/si-Nodal nanocomplexes inhibited the proliferation and metastasis of CRC in vitro.**

A–B. Colony formation and CCK8 assays confirmed the decreased proliferation ability of cells treated with BSA-NP/si-Nodal.

C–D. Transwell assay confirmed the decreased migration and invasion abilities of cells treated with BSA-NP/si-Nodal.

E. H&E staining of the major organs in mice with different treatments.

F-G. CCK-8 and colony formation assays confirmed the SCD1 overexpression rescued the decreased proliferation ability of cells treated with BSA-NP/si-Nodal.

H. Transwell assay confirmed the SCD1 overexpression rescued the decreased migrative and invasive ability of cells treated with BSA-NP/si-Nodal.

I. Western blot confirmed the SCD1 overexpression rescued the EMT inhibition of cells treated with BSA-NP/si-Nodal.

(∗∗∗P < 0.001)

| **Table S1. PCR primer, siRNA and plasmid sequence.** | |
| --- | --- |
| **Primes** | **5’-3’ sequence** |
| **Primers for PCR** |  |
| Nodal-forward | AGAAGCAGATGTCCAGGGTAGC |
| Nodal-reverse | AGAGGCACCCACATTCTTCC |
| SCD1-forward | CTTGACACAGGTGCCATC |
| SCD1-reverse | GGGGGCTAATGTTCTTGTCA |
| GAPDH-forward | CAGGAGGCATTGCTGATGAT |
| GAPDH-reverse | GAAGGCTGGGGCTCATTT |
| **siRNAs** |  |
| Si-nodal#1 | UCAAGUUCCAGGUGGACUUTT |
| Si-nodal#2 | GCGAGUGUCCUAAUCCUGUTT |
| Si-nodal#3 | GCUCCUAGAUCACCAUAAATT |
| Si-SCD1 | GCATTCCAGAATGATGTCTAT |
| Control | UUCUCCGAACGUGUCACGUTT |
| **Primers for ChIP-qPCR** |  |
| SCD1 promoter-forward-1 | GAGCCAGGACTGGAATGC |
| SCD1 promoter-reverse-1 | GTGCTGCGCCAATGATTC |
| SCD1 promoter-forward-2 | CCAAGCTCCAGATCCTGG |
| SCD1 promoter-reverse-2 | CACCACTAACATCTCCGTCC |
